# Neoadjuvant Modified Short-Course Radiotherapy Followed by Delayed Surgery for Locally Advanced Rectal Cancer

**DOI:** 10.3390/cancers13164112

**Published:** 2021-08-15

**Authors:** Hiroshi Doi, Hiroyuki Yokoyama, Naohito Beppu, Masayuki Fujiwara, Shogo Harui, Ayako Kakuno, Hidenori Yanagi, Yoshio Hishikawa, Naoki Yamanaka, Norihiko Kamikonya

**Affiliations:** 1 Department of Radiation Oncology, Meiwa Cancer Clinic, 3-39 Agenaruocho, Hyogo, Nishinomiya 663-8186, Japan; h-yokoyama@hyo-med.ac.jp (H.Y.); m-fuji@hyo-med.ac.jp (M.F.); harui.shogo@gmail.com (S.H.); hishikawa-yoshio@medipolis.org (Y.H.); kamikonya.n@meiwa-hospital.com (N.K.); 2 Department of Radiation Oncology, Kindai University Faculty of Medicine, 377-2 Ohno-Higashi, Osaka-Sayama, Osaka 589-8511, Japan; 3 Department of Radiology, Hyogo College of Medicine, 1-1 Mukogawa-cho, Hyogo, Nishinomiya 663-8501, Japan; 4 Department of Surgery, Meiwa Hospital, 4-31 Agenaruo, Hyogo, Nishinomiya 663-8186, Japan; beppu-n@hyo-med.ac.jp (N.B.); yanagih@meiwa-hospital.com (H.Y.); nyamana@meiwa-hospital.com (N.Y.); 5 Division of Lower Gastrointestinal Surgery, Department of Surgery, Hyogo College of Medicine, 1-1 Mukogawa-cho, Hyogo, Nishinomiya 663-8501, Japan; 6 Department of Pathology, Meiwa Hospital, 4-31 Agenaruo, Hyogo, Nishinomiya 663-8186, Japan; sugihara@meiwa-hospital.com

**Keywords:** rectal cancer, accelerated hyperfractionated radiotherapy, preoperative radiotherapy, neoadjuvant radiotherapy, short-course radiotherapy, capecitabine, S-1, neutrophil-to-lymphocyte ratio, chemotherapy, radiotherapy

## Abstract

**Simple Summary:**

Both short- and long-course neoadjuvant radiotherapy (NA-RT) followed by surgery have been adopted as standard treatments for locally advanced rectal cancer (LARC). We hypothesized that a modified short-course radiotherapy (mSC-RT) using an accelerated hyperfractionated regimen, with a dose of 2.5 Gy twice daily up to a total dose of 25 Gy in 10 fractions, can provide a favorable therapeutic ratio in comparison with the conventional regimens. Ninety-seven consecutive LARC patients undergoing mSC-RT followed by delayed surgery were analyzed in this retrospective study. Additionally, potential prognostic factors for overall survival (OS) were also assessed. The results showed that mSC-RT followed by delayed surgery achieved equivalent anti-tumor efficacy and acute toxicity that were comparable with long- and short-course NA-RT, respectively. A neutrophil-to-lymphocyte ratio (NLR) ≥ 1.83 was independently associated with poor OS in LARC patients receiving mSC-RT. Thus, mSC-RT can be a promising alternative to both standard long- and short-course NA-RT regimens.

**Abstract:**

This study aimed to assess the clinical outcomes and predictive factors of neoadjuvant modified short-course radiotherapy (mSC-RT) for locally advanced rectal cancer (LARC). Data from 97 patients undergoing mSC-RT followed by radical surgery for LARC were retrospectively analyzed. A 2.5 Gy dose twice daily up to a total dose of 25 Gy in 10 fractions was administered through mSC-RT, and this was delivered with oral chemotherapy in 95 (97.9%) patients. Radical surgery was performed 6 (range, 3–13) weeks after mSC-RT. The median follow-up among surviving patients was 43 (8–86) months. All patients completed neoadjuvant radiotherapy with no acute toxicity grade ≥ 3. Three- and five-year local control rates were 96.3% and 96.3%, respectively. Three- and five-year overall survival (OS) rates were 92.7% and 79.8%, respectively. Univariate analyses revealed that poor OS was associated with no concurrent administration of capecitabine, C-reactive-protein-to-albumin ratio ≥ 0.053, carcinoembryonic antigen ≥ 3.4 ng/mL, and neutrophil-to-lymphocyte ratio (NLR) ≥ 1.83 (P = 0.045, 0.001, 0.041, and 0.001, respectively). Multivariate analyses indicated that NLR ≥ 1.83 was independently associated with poor OS (*p* = 0.018). mSC-RT followed by delayed surgery for LARC was deemed feasible and resulted in good clinical outcomes, whereas poor OS was associated with high NLR.

## 1. Introduction

Neoadjuvant radiotherapy (NA-RT) combined with chemotherapy and total mesorectal excision has been adopted as the standard treatment for locally advanced rectal cancer (LARC). Two different approaches of NA-RT are commonly used for LARC: short-course NA-RT (25 Gy in five fractions) and long-course NA-RT (45 to 50.4 Gy in 25 to 28 fractions) [1,2,3,4]. The Polish trial and the TROG 01.04 trial have revealed that there are no significant differences in overall survival (OS), local control (LC), distant failure, relapse-free survival (RFS), and late toxicities in randomized controlled trials comparing the short- and long-course NA-RT [5,6]. Moreover, meta-analyses have also shown that there are no significant differences in LC between short-course and long-course NA-RT [7,8].

Short-course NA-RT has several features that are different from those of long-course NA-RT. The efficacy of consolidation chemotherapy following short-course NA-RT is controversial [9]. However, the RAPIDO trial showed that short-course NA-RT had a lower disease-related treatment failure rate than long-course NA-RT when chemotherapy was delivered following NA-RT prior to surgery in both groups [10]. In terms of the interval between short-course NA-RT and surgery, the Stockholm III randomized study compared preoperative short-course RT followed by immediate or delayed surgery and showed a significantly higher pathological complete response rate in the delayed surgery group with an interval of 4–8 weeks than in the immediate surgery group [11]. They also showed that the postoperative complication rate was significantly lower in the delayed surgery group [12]. Therefore, short-course radiotherapy with delayed surgery has been considered to be a feasible alternative to short-course NA-RT with immediate surgery and long-course NA-RT.

The conventional short-course NA-RT regimen of 25 Gy/5 fractions is reportedly associated with a higher incidence of surgical complications because of the high dose of radiotherapy used per fraction [13]. Delayed surgery could be considered as an option to increase the feasibility of short-course NA-RT. An accelerated hyperfractionated regimen of radiotherapy has been a prospective approach to separate the biologically equivalent dose (BED) between the normal tissue and the tumor and to reduce radiation-induced damages to normal tissues while keeping therapeutic efficacy against cancer cells [14]. Therefore, we hypothesized that short-course NA-RT using an accelerated hyperfractionated regimen for LARC could be highly effective and well-tolerated when compared to conventional short-course NA-RT.

The addition of chemotherapy to long-course NA-RT has been demonstrated to be beneficial, with enhanced tumoricidal effects [15]. The use of 5-fluorouracil (5-FU)-based chemotherapy has gained widespread acceptance for the treatment of LARC [1,2,3,4]. Oral fluoropyrimidines, such as capecitabine and S-1, are generally more convenient for patients compared to 5-FU infusions. We have previously reported the tolerability and efficacy of modified short-course radiotherapy (mSC-RT) using an accelerated hyperfractionated regimen that was combined with such oral chemotherapy along with clinical features [16,17,18].

Prognostic factors in cancer treatment can provide significant information for clinicians and patients to make an appropriate decision when choosing a treatment modality. Several host factors and serum factors have been assessed in rectal cancer [19,20,21,22]. The systemic inflammatory response is involved in the progression, treatment response, and prognosis after treatment [20,21,22]. This systemic inflammatory response can be reflected by hematological parameters such as the neutrophil-to-lymphocyte ratio (NLR) and C-reactive-protein-to-albumin ratio (CAR), which can be a predictor for oncological outcomes [21,22]. However, there are only a few reports showing predictive factors after short-course NA-RT for LARC.

The purpose of this study was to update the clinical outcomes of neoadjuvant mSC-RT for LARC and assess the predictive factors after mSC-RT.

## 2. Materials and Methods

This study was approved by our institutional review board with approval number 2020–26.

### 2.1. Patients

A total of 141 patients with primary rectal adenocarcinoma who underwent NA-RT at Meiwa Cancer Clinic (Hyogo, Japan) between April 2014 and March 2020 were identified in our prospective database. We confirmed the clinical stage at the initial diagnosis for this study based on the 8th edition of the International Union Against Cancer (UICC)/American Joint Committee on Cancer (AJCC) tumor-node-metastasis (TNM) classification system [23]. We excluded the following patients: 8 and 20 patients who had Stage I and IV rectal cancer, respectively; 7 patients who received NA-RT in a conventional fraction; 5 patients who did not undergo surgical resection at our institute; a patient who had a previous irradiation procedure done in the pelvis; and 3 patients who had a follow-up duration of less than 6 months without any specific events. Data from the 97 remaining patients with stage II or III rectal cancer who underwent NA-RT followed by radical surgery at Meiwa Hospital (Hyogo, Japan) were retrospectively analyzed in this study. The patient characteristics are summarized in Table 1.

### 2.2. Treatment

For radiotherapy, all eligible patients were placed in the supine position and were helically scanned using an Aquilion LB (Canon Medical Systems, Japan) CT unit. For each patient, a planning CT scan of the entire pelvis from the lower-abdomen down until below the ischial tuberosities was obtained at 5-mm intervals. The CT data sets from the FOCUS XiO™ (Elekta AB, Stockholm, Sweden) between April 2014 and June 2019 and from Monaco version 5.11.03 (Elekta AB, Stockholm, Sweden) from July 2019 onwards were transferred to the treatment planning system to outline the volumes of interest.

The gross target volume (GTV) included the primary rectal tumor and the nodal metastasis, whereas the clinical target volume (CTV) contained the GTV with a 0.5 cm margin, as well as the perirectal, presacral, and internal iliac nodes. The planning target volume (PTV) was the CTV with a 0.5 cm margin. Furthermore, there was an additional 7 mm leaf margin added to the PTV in order to cover the PTV more homogenously. The field margins of each beam were defined and expanded as follows: the cranial margins were the anterior iliac crests or the L4–5 interspace, the caudal margins were the ischial tuberosities, the lateral margins were expanded 1.5 cm beyond the sacroiliac joint, the anterior margins were the dorsal edge of the pubic joint, and the posterior field margins were designed to include the posterior edge of the sacrum.

Radiotherapy was performed using a three-dimensional conformal technique, which was typically carried out with a 4-field box technique using 10 MV photons. The planned radiotherapy was delivered using an Elekta Synergy (Elekta AB, Stockholm, Sweden) unit.

The patients were treated with a dose of 2.5 Gy twice daily, with an interval of at least 6 h between fractions, up to a total dose of 25 Gy in 10 fractions over one week. The protocol recommended a treatment time of 5 consecutive days from Monday to Friday. S-1 (Taiho Pharmaceutical Co., Tokyo, Japan; 60 mg/m^2^/day) between April 2014 and March 2015 or capecitabine (Xeloda; Roche Ltd., Basel, Switzerland; 825 mg/m^2^/day) from April 2015 onwards was typically administered orally together with NA-RT. Unless there was lateral lymph nodal metastasis, total mesorectal excision (TME) without prophylactic lateral lymph node dissection was performed 4 weeks after radiotherapy.

### 2.3. Analysis

The data of continuous variables are expressed as medians, with the range in the parentheses, unless otherwise indicated. The time-to-outcome in our study was considered from the start of the radiotherapy to the data of a specific event. Local recurrence was defined as evidence of recurrent disease within the pelvis after a surgical resection. The reported events for RFS were: local recurrence, distant failure, and death. Toxicity was evaluated using the Common Terminology Criteria for Adverse Events (CTCAE) version 5.0 [24].

The pathological tumor response after NA-RT was determined according to the following pathological grading: Grade 0, not effective; grade 1a, high response in <1/3 of the cancer cells; grade 1b, high response in 1/3–2/3 of the cancer cells; grade 2, high response in >2/3 of the cancer cells; and grade 3, complete response [25]. In addition, patients with grades 2 or 3 tumor response were defined as responders, whereas patients with grades 0, 1a, or 1b tumor response were defined as non-responders.

Time-to-outcome Kaplan–Meier curves were depicted, and the differences in selected populations were analyzed through the log-rank test. The Cox proportional-hazards model was used to evaluate factors that influence OS. The cut-off values of possible serum predictive factors were decided based on the receiver operating characteristic (ROC) curves. The results were reported as hazard ratios with corresponding 95% confidence intervals (CI). Variables with *p*-values of <0.20 according to univariate analysis were analyzed in the multivariate model with Cox regression analysis. The Wilcoxon test was used to compare continuous variables and trends among groups. All statistical analyses were performed using the GraphPad Prism version 8.4.3 (GraphPad Software, Inc., San Diego, CA, USA). The JMP software version 12.2.0 (SAS Institute, Cary, NC, USA) was used to perform Cox regression analysis, and *p*-values of <0.05 were considered as statistically significant.

## 3. Results

The median follow-up in all eligible and alive patients was 41 (1–86) and 43 (8–86) months, respectively.

All patients completed NA-RT within 7 days with no cessation. The acute adverse events before surgery are listed in Table 2. No grade ≥ 3 toxicity was observed before surgery.

Radical surgery was performed 6 (3–13) weeks after radiotherapy. The sphincter-saving resection was performed in 90 patients (92.8%), including one patient who received partial excision surgery. Among 74 patients with lower rectal cancer (Rb), 69 patients (93.2%) received sphincter-saving surgery. Adjuvant chemotherapy was administered in 37 patients (38.1%). Except for two patients with pathologically positive surgical margin (R1), complete (R0) resection of the primary tumor was achieved in 95 (97.9%) patients. Additionally, peritoneal dissemination was found intraoperatively in two patients. All four patients who had possible residual cancer cells underwent adjuvant chemotherapy. Pathological responses to neoadjuvant chemoradiotherapy and the stages are shown in Table 3. Pathological complete response was observed in 13 (14.1%) patients.

Temporary stoma closing surgery was performed in 80 patients 14 (6–59) weeks after radical surgery, and the stoma was reconstructed in two of them due to pain and fistula formation 13 and 11 months after colostomy closure, respectively. Therefore, 79 of all eligible patients (81.4%) and 58 patients in the Rb LARC group (78.4%) could pass material through their anus. Perioperative complications are shown in Table 4. Perioperative complications grade ≥ 3 were observed in 11 patients (11.3%), and one patient experienced aspiration pneumonia resulting in multiple organ failure.

**Figure 1:**
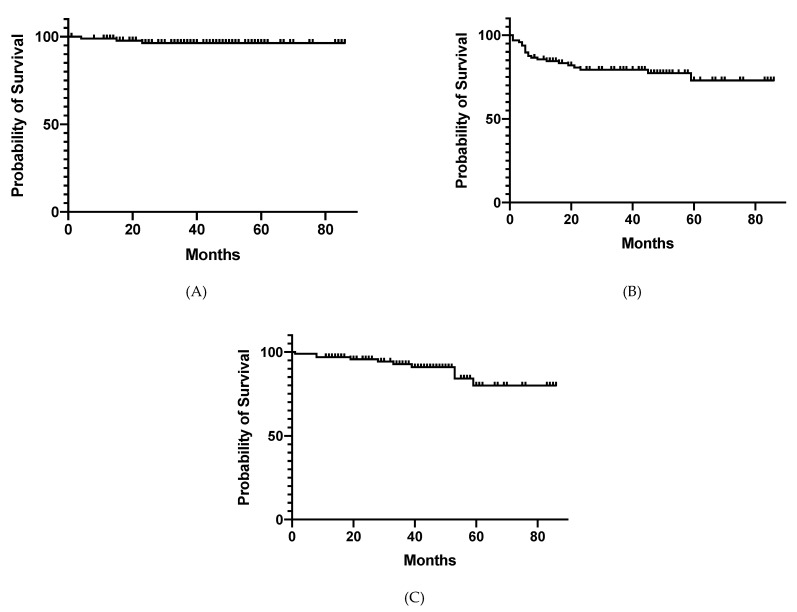
Clinical outcomes after neoadjuvant radiotherapy. Cumulative rates of local control, relapse-free survival, and overall survival are shown in (**A**), (**B**), and (**C**), respectively.

Three patients developed intra-pelvic failure during the follow-up term. The 1-, 3-, and 5-year LC rates were 99.0%, 96.3%, and 96.3%, respectively (Figure 1A). Seventeen patients developed out-of-pelvic failures, including liver metastases, lung metastases, para-aortic nodal metastases, peritoneal dissemination, kidney metastasis, uterine metastasis, and bone metastasis in 11, 8, 3, 1, 1, 1 and 1 patient(s), respectively. Twelve patients died during the follow-up term. Two patients died of small cell lung cancer and pancreatic cancer 45 and 59 months after NA-RT, respectively. The 1-, 3-, and 5-year RFS rates were 85.6%, 79.3%, and 72.9%, respectively (Figure 1B). The 1-, 3-, and 5-year OS rates were 96.9%, 92.7%, and 79.8%, respectively (Figure 1C).

**Figure 2:**
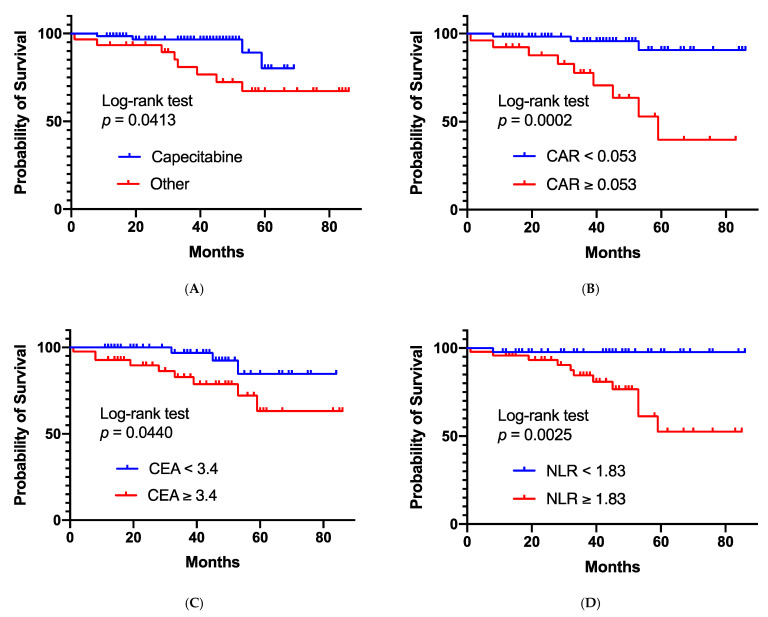
Overall survivals per each risk factor. Cumulative rates of overall survival for each risk factor, which were identified in univariate analyses using Cox regression analysis. Poor overall survival was significantly associated with no concurrent administration of capecitabine (**A**), high C-reactive-protein-to-albumin ratio (CAR) (**B**), high carcinoembryonic antigen (CEA) (**C**), and high neutrophil-to-lymphocyte ratio (NLR) (**D**).

The ROC curve indicated cut-off values of 0.053, 3.4, and 1.83 for C-reactive-protein-to-albumin ratio (CAR), carcinoembryonic antigen (CEA), and neutrophil-to-lymphocyte ratio (NLR), respectively (Supplemental Figure S1). Univariate analyses revealed that the following factors were significantly associated with poor OS: no concurrent administration of capecitabine, high CAR, high CEA level, and high NLR (Table 5, Figure 2). Multivariate analysis indicated that high NLR was independently associated with poor OS.

Late postoperative adverse events are shown in Table 6. Grade 3 late adverse events were observed in 10 patients (10.3%).

## 4. Discussion

In this study, we presented the clinical outcomes of mSC-RT for LARC along with the details of the adverse events and possible predictive factors. Based on the results, mSC-RT was demonstrated to be highly feasible, achieving an excellent complete resection rate and local control rate and a reasonable survival rate. Further, NLR ≥ 1.83 was revealed as an independent predictor for poor OS in this study.

A multimodal approach has become the standard of care for LARC. In addition, the short-course NA-RT regimen of 25 Gy/5 fractions is one of the most common regimens [1,2,3,4,5,6,7,8,9,10,11,12,13]. The mSC-RT using an accelerated hyperfractionated regimen seems to be a treatment regimen with a favorable therapeutic ratio, with a high BED calculation for tumor in comparison with the conventional short-course NA-RT regimen [14]. The value of α/β was 10 Gy for the rectal tumor. For tumor effects, the BED of the mSC-RT was 31.3 Gy using the formula: BED = nd [1 + (d/α/β)] − γ/α (T–Tk), where *n* is the number of fractions and d is the single fraction dose. Viani et al. have reported in a systematic review that NA-RT with a BED > 30 Gy significantly improved the local control for rectal cancer [26]. Thus, the BED of the present mSC-RT seems to be able to provide the equivalent efficacy for LARC.

The regimen of mSC-RT was combined with oral administration of chemotherapy in most cases in this study. Chemotherapy agents of choice were S-1 from 2014 to 2017 and capecitabine from 2017 onwards due to institutional protocol. The additional benefit of concurrent chemotherapy over short-course NA-RT was proven in only a limited number of prospective randomized trials and is still controversial at present. By itself, mSC-RT may be underpowered in terms of therapeutic efficacy, and thus we hypothesized that oral chemotherapy could enhance the therapeutic benefit of NA-RT for LARC. Response to the conventional NART, followed by surgery weeks later, varied between patients. Complete resection rate, pathological complete response rate, 5-year local control rate, and overall survival rate have been reported to be 90–95%, 10–20%, >90%, and 60–85%, respectively [27,28]. In addition, the incidence of perioperative complications and a grade 3 late toxicity rate of approximately 10% were also consistent with those described in previous reports [5,6,29]. Furthermore, excellent sphincter preservation rate was also demonstrated in this study [30]. Taken together, mSC-RT followed by delayed surgery achieved equivalent anti-tumor efficacy that was comparable with long-course NA-RT and an acceptable toxicity profile. Notably, acute toxicity was comparable with conventional short-course NA-RT, which can be significantly milder than long-course NA-RT. This would be less likely to interfere in the concurrent systemic therapy and can benefit the patients’ convenience [29]. In addition, NA-RT has recently been recommended in management of elderly patients with LARC [31]. mSC-RT seems considerable in various scenarios because the mild toxicities before surgery were observed in this study. Therefore, we believe mSC-RT can be a promising alternative to both standard long- and short-course NA-RT regimens.

We examined several prognostic factors in this study and observed that poor OS was associated with no concurrent administration of capecitabine, high CAR, high CEA level, and high NLR in univariate analyses. The findings in these serum markers were consistent with previous reports. The use of capecitabine led to a longer survival in comparison with other chemotherapeutic agents or with radiation alone in the Cox hazard model. However, no significant differences were observed between capecitabine and S-1. It has been reported that capecitabine is a recommended agent in combined use with NA-RT [4]. Allegra et al. have demonstrated that the neoadjuvant use of capecitabine, when combined with radiation therapy, is comparable with continuous 5-FU infusion in patients with stage II or III rectal cancer in a phase III randomized trial [32]. Comparing capecitabine and S-1, a phase III randomized trial showed that there were no significant differences in progression-free survival and OS in metastatic colorectal cancers [33]. Sadahiro et al. have reported that the efficacy of S-1 or capecitabine combined with NA-RT for LARC seems to be equivalent [34]. Furthermore, capecitabine was used more recently than S-1 at our institute. This, together with the sophistication of the medical team, supportive care, and systemic chemotherapy, might benefit the OS in patients receiving capecitabine.

We presented that NLR with a cut-off value of 1.83 was a significant predictor for OS in this study. NLR, an inflammatory and immune factor, has been reported as a predictor of OS in various solid tumors, including rectal cancer [21]. However, to the best of our knowledge, there are only a few previous reports indicating the utility of NLR in LARC patients undergoing short-course NA-RT, including mSC-RT combined with chemotherapy. Lymphocytes initially provide protection against cancer cell proliferation and migration. Activated T-cells can be suppressed by marked neutrophil infiltration, and a high NLR could decrease the effects of the lymphocyte-mediated cellular immune response, which could promote cancer progression [20]. Therefore, the present data are consistent with the previous reports that patients with low NLR developed better survival outcomes than those with high NLR. Additionally, a low-range cut-off value (1.83) was obtained in this study. Furthermore, previous reports have indicated that the NLR might predict the effects of immune checkpoint inhibitors in patients with various malignancies [35]. Thus, we hypothesize that patients with high NLR could be candidates for adjuvant chemotherapy and/or target therapy rather than immune checkpoint inhibitors.

We acknowledge that there are several limitations to this study. We presented good outcomes after mSC-RT combined with oral chemotherapy for LARC in a limited number of patients with a relatively short follow-up. However, we were able to present the patients’ background, along with the results of the multivariate analysis. In addition, the median follow-up was longer than 3 years, which can be sufficient to evaluate the short-term outcomes.

Some notable types of modern surgical procedures including robotic surgery and transanal TME (TaTME) have been described [36,37]. Robotic surgery enables highly flexible and accurate surgery and is expected to improve outcomes over standard laparoscopic surgeries, especially for ultra-low rectal cancer. A meta-analysis has shown that robotic surgery can reduce conversion rates to open surgery, in comparison with laparoscopic surgery [36]. Rectal surgery can be associated with damage to the pelvic organs, leading perioperative complications, and late toxicities, as we described in this study (Table 4 and Table 6). TaTME has been described in challenging cases with the aim of radical resection, the preservation of pelvic nerves, and the achievement of a restorative procedure [37]. Preferable surgical procedures following mSC-RT should be investigated and might improve clinical outcomes, although we demonstrated favorable therapeutic outcomes along with a high tolerability in this study.

Based on the present study, we believe that mSC-RT combined with oral chemotherapy could be an alternative to the conventional long- and short-course NA-RT for LARC. Longer follow-up and a prospective controlled study should be performed in the future in order to clarify the efficacy of mSC-RT.

## 5. Conclusions

To conclude, we herein presented that mSC-RT for LARC was well tolerated by the patients and produced excellent clinical outcomes. Poor OS was associated with high NLR. As such, mSC-RT can be a promising alternative to conventional long- and short-course NA-RT regimens. Further trials with larger numbers of homogenous patients with LARC and longer follow-up periods are warranted based on our findings.

## Figures and Tables

**Table 1:** Patient characteristics.

Characteristics		*n* = 97	(%)
Age, years (range)		66 (34–87)	
Sex	Male	52	(53.6)
	Female	45	(46.4)
ECOG-PS	0	80	(82.5)
	1	15	(15.5)
	2	1	(1.0)
	3	1	(1.0)
Localization	Ra	23	(23.7)
	Rb	74	(76.3)
Pathological diagnosis			
	Tubular adenocarcinoma	92	(94.8)
	Papillary adenocarcinoma	3	(3.1)
	Poorly differentiated adenocarcinoma	2	(2.1)
Clinical stage at the diagnosis			
T	1	3	(3.1)
	2	31	(32.0)
	3	50	(51.5)
	4	13	(13.4)
N	0	8	(8.2)
	1	53	(54.6)
	2	36	(37.1)
Stage	II	9	(9.3)
	III	88	(90.7)
Previous chemotherapy before radiotherapy ^†^			
	Any	34	(35.1)
	None	63	(64.9)
Combined chemotherapy			
	Capecitabine	67	(69.1)
	S-1	22	(22.7)
	UFT	5	(5.2)
	Polysaccharide-K	1	(1.0)
	none	2	(2.1)
Adjuvant chemotherapy after surgery			
	Any	37	(38.1)
	None	60	(61.9)

^†^ Previous chemotherapy included chemotherapy which was performed more than 30 days before radiotherapy. Abbreviations: ECOG-PS, Eastern Cooperative Oncology Group performance status.

**Table 2:** Adverse events before surgery.

Adverse events per patient		*n* = 97	Incidence (%)
Any		16	(16.5)
	Worst grade per patient	*n* = 97	Incidence (%)
Grade	1	13	(13.4)
	2	3	(3.1)
None		81	(83.5)
Adverse events	Grade	*n* = 97	Incidence (%)
Nausea	1	4	(4.1)
	2	1	(1.0)
Diarrhea	1	5	(5.2)
Enterocolitis	1	2	(2.1)
	2	1	(1.0)
Fatigue	1	3	(3.1)
Dermatitis	2	1	(1.0)
Anorexia	1	1	(1.0)
Urinary frequency	1	1	(1.0)
Cystitis noninfective	1	1	(1.0)

**Table 3:** Pathological responses to neoadjuvant therapy.

Pathological tumor response		*n* = 92 *	(%)
Grade	0	1	(1.1)
	1a	21	(22.8)
	1b	22	(23.9)
	2	35	(38.0)
	3	13	(14.1)
Pathologic stage			
T	0	13	(13.4)
	Tis	3	(3.1)
	1	14	(14.4)
	2	27	(27.8)
	3	33	(34.0)
	4	7	(7.2)
N	0	62	(63.9)
	1	25	(25.8)
	2	10	(10.3)
Stage	0	15	(15.5)
	I	32	(33.0)
	II	15	(15.5)
	III	33	(34.0)
	IV	2	(2.1)

* Excluding five patients in whom pathological grades were not available.

**Table 4:** Perioperative complications.

Adverse events per patient		*n* = 97	Incidence (%)
Any		50	(51.5)
	Worst grade per patient	*n* = 97	Incidence (%)
Grade	1	5	(5.2)
	2	34	(35.1)
	3	10	(10.3)
	4	0	(0.0)
	5	1	(1.0)
None		47	(48.5)
Adverse events	Grade	*n* = 97	Incidence (%)
Ileus	1	1	(1.0)
	2	11	(11.3)
	3	4	(4.1)
Dysuria	1	1	(1.0)
	2	5	(5.2)
Fever	1	6	(6.2)
Pelvic infection	2	4	(4.1)
	3	1	(1.0)
Rectal anastomotic leak	2	2	(2.1)
	3	2	(2.1)
Colonic fistula	2	1	(1.0)
	3	2	(2.1)
Colonic obstruction	2	3	(3.1)
Intestinal stoma obstruction	2	3	(3.1)
Dehydration	2	2	(2.1)
Nausea	2	2	(2.1)
Wound infection	2	2	(2.1)
Abdominal infection	2	2	(2.1)
Fatigue	2	1	(1.0)
	3	1	(1.0)
Rectal stenosis	1	1	(1.0)
	3	1	(1.0)
Aspiration	5	1	(1.0)
Prolapse of intestinal stoma	3	1	(1.0)
Postoperative hemorrhage	3	1	(1.0)
Enterocolitis	2	1	(1.0)
Enterocolitis infectious	2	1	(1.0)
Anal pain	2	1	(1.0)
Stoma site infection	2	1	(1.0)
Intestinal stoma leak	2	1	(1.0)
Large intestinal anastomotic leak	2	1	(1.0)
Lymphedema	2	1	(1.0)
Stomal ulcer	2	1	(1.0)
Diarrhea	2	1	(1.0)
Chylous ascites	1	1	(1.0)

**Table 5:** Univariate and multivariate analyses for the factors associated with overall survival.

		Univariate Analysis		Multivariate Analysis	
Factors	*n* = 97	Hazard Ratio (95% CI)	*p*-Value	Hazard Ratio (95% CI)	*p*-Value
Age (y)					
< 65	48	1	0.191	1	0.865
≥ 65	49	2.177 (0.684–8.168)		1.137 (0.256–5.358)	
Sex					
Male	52	1	0.309		
Female	45	1.805 (0.576–6.103)			
ECOG-PS					
0	80	1	0.378		
1-	17	0.440 (0.024–2.264)			
Location of primary tumor					
Ra	23	1	0.456		
Rb	74	1.725 (0.454–11.226)			
Primary tumor stage					
cT1-3	84	1	0.303		
cT4	13	2.083 (0.462–7.000)			
cStage					
2	9	1	0.093	1	0.306
3	88	not applicable		not applicable	
Previous chemotherapy before neoadjuvant radiotherapy					
Yes	34	1	0.466		
No	63	0.647 (0.206–2.193)			
Chemotherapy administered with radiotherapy					
Capecitabine	67	1	0.045	1	0.223
Other	30	3.310 (1.027–12.538)		2.598 (0.574–14.103)	
Capecitabine	67	1	0.132		
S-1	23	2.667 (0.741–10.655)			
Adjuvant chemotherapy					
Yes	37	1	0.777		
No	60	1.189 (0.373–4.469)			
Residual tumor condition after surgery					
R0 resection	93	1	0.084	1	0.443
R1 or presence of dissemination	4	4.988 (0.765–19.004)		2.212 (0.249–14.846)	
Sphincter preservation surgery					
Yes	90	1	0.230		
No	7	not applicable			
Interval between neoadjuvant radiotherapy and surgery					
< 6 weeks	36	1	0.165	1	0.378
≥ 6 weeks	61	0.432 (0.113–1.404)		2.082 (0.396–10.681)	
Pathological response to the preoperative treatment	*n* = 92				
Responder	48	1	0.806		
Non-responder	44	1.153 (0.360–3.696)			
Pathological findings					
Complete response	13	1	0.447		
Other	84	2.054 (0.398–37.583)			
C- reactive-protein-to-albumin ratio	*n* = 85				
<0.053	59	1	0.001	1	0.157
≥0.053	26	8.103 (2.407–36.632)		2.801 (0.681–14.356)	
CEA	*n* = 87				
<3.4 ng/mL	46	1	0.041	1	0.400
≥3.4 ng/mL	41	3.522 (1.050–15.885)		1.819 (0.461–8.924)	
Neutrophil-to-lymphocyte ratio	*n* = 90				
<1.83	43	1	0.001	1	0.018
≥1.83	47	12.054 (2.328–220.775)		8.682 (1.376–175.625)	

Abbreviations: CI, confidence interval; ECOG-PS, Eastern Cooperative Oncology Group performance status; CEA, carcinoembryonic antigen.

**Table 6:** Postoperative late adverse events.

Adverse events per patient		*n* = 97	Incidence (%)
Any		45	(46.4)
	Worst grade per patient	*n* = 97	Incidence (%)
Grade	1	12	(12.4)
	2	23	(23.7)
	3	10	(10.3)
None		47	(48.5)
Adverse events	Grade	*n* = 97	Incidence (%)
Diarrhea	1	13	(13.4)
	2	10	(10.3)
Ileus	1	1	(1.0)
	2	3	(3.1)
Colonic obstruction	2	2	(2.1)
	3	1	(1.0)
Wound dehiscence	2	1	(1.0)
	3	2	(2.1)
Pelvic infection	2	1	(1.0)
	3	2	(2.1)
Constipation	1	2	(2.1)
	2	1	(1.0)
Gastrointestinal fistula	3	2	(2.1)
Vascular access complication	3	1	(1.0)
Gastrointestinal disorders—other, specify (mucosal prolapse)	3	1	(1.0)
Anal pain	3	1	(1.0)
Enterocolitis	2	1	(1.0)
Rectal anastomotic leak	2	1	(1.0)
Erectile dysfunction	2	1	(1.0)
Cystitis noninfective	2	1	(1.0)
Rectal stenosis	2	1	(1.0)
Colonic fistula	1	1	(1.0)
Fatigue	1	1	(1.0)
Anal pain	1	1	(1.0)

## Data Availability

The data are available from the corresponding authors upon reasonable request.

## Supplementary Materials

The following are available online at https://www.mdpi.com/article/10.3390/cancers13164112/s1, Figure S1: Receiver operating characteristic (ROC) curves to identify cut-off values for possible serum predictive factors. ROC curves were generated to identify cut-off values for possible serum predictive factors including C-reactive protein to albumin ratio (CAR) (A), carcinoembryonic antigen (CEA) (B), and neutrophil-to-lymphocyte ratio (NLR) (C). Area under the ROC curve was 0.71, 0.66, and 0.68 in A, B, C, respectively.
