# Supplementary material for: Neoadjuvant Modified Short-Course Radiotherapy Followed by Delayed Surgery for Locally Advanced Rectal Cancer
Source: Cancers (Basel). 2021 Aug 15;13(16):4112. doi: 10.3390/cancers13164112 (PMC8394890; doi:10.3390/cancers13164112)
Supplement: Supplementary file 1 [file cancers-13-04112-s001.zip › cancers-1329041-supplementary.pdf]

## Supplemental Figure S1

A

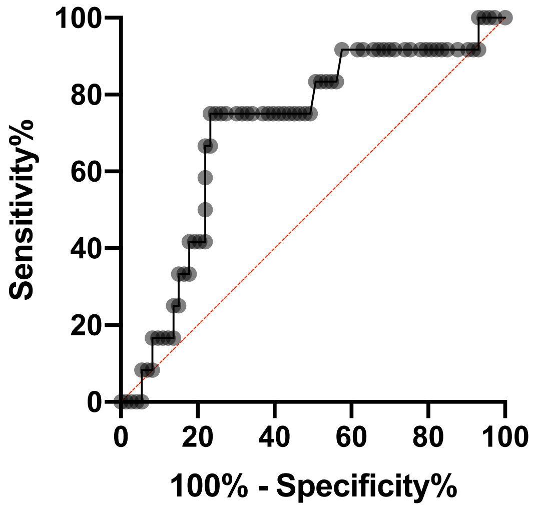


B

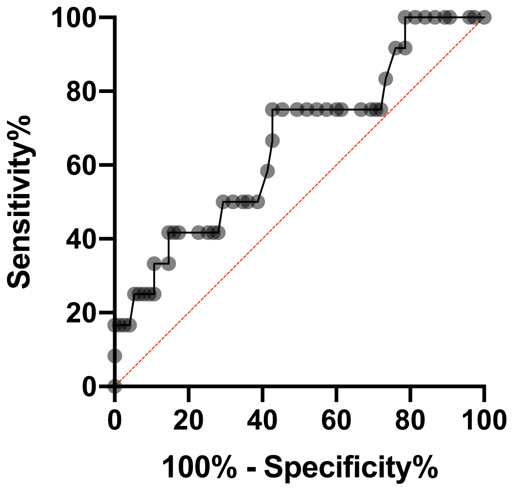


C

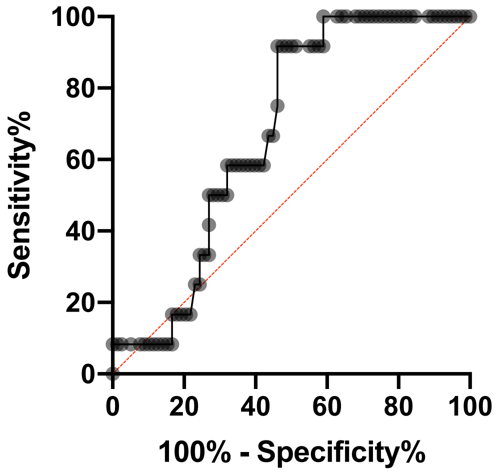


**Figure S1:** Receiver operating characteristic (ROC) curves to identify cut-off values for possible serum predictive factors. ROC curves were generated to identify cut-off values for possible serum predictive factors including C-reactive protein to albumin ratio (CAR) (A), carcinoembryonic antigen (CEA) (B), and neutrophil-to-lymphocyte ratio (NLR) (C). Area under the ROC curve was 0.71, 0.66, and 0.68 in A, B, C, respectively.
